# Liposome-Mediated Herpes Simplex Virus Uptake Is Glycoprotein-D Receptor-Independent but Requires Heparan Sulfate

**DOI:** 10.3389/fmicb.2016.00973

**Published:** 2016-06-22

**Authors:** Lorrie A. Burnham, Dinesh Jaishankar, Jeffrey M. Thompson, Kevin S. Jones, Deepak Shukla, Vaibhav Tiwari

**Affiliations:** ^1^Department of Biology, California State UniversitySan Bernardino, CA, USA; ^2^Departments of Ophthalmology and Visual Sciences, Bioengineering and Microbiology/Immunology, University of IllinoisChicago, IL, USA; ^3^Department of Biology, Howard UniversityWashington, DC, USA; ^4^Department of Microbiology and Immunology, Midwestern UniversityDowners Grove, IL, USA

**Keywords:** heparan sulfate, virus-cell interactions, viral entry

## Abstract

Cationic liposomes are widely used to facilitate introduction of genetic material into target cells during transfection. This study describes a non-receptor mediated herpes simplex virus type-1 (HSV-1) entry into the Chinese hamster ovary (CHO-K1) cells that naturally lack glycoprotein D (gD)-receptors using a commercially available cationic liposome: lipofectamine. Presence of cell surface heparan sulfate (HS) increased the levels of viral entry indicating a potential role of HS in this mode of entry. Loss of viral entry in the presence of actin de-polymerizing or lysosomotropic agents suggests that this mode of entry results in the endocytosis of the lipofectamine-virus mixture. Enhancement of HSV-1 entry by liposomes was also demonstrated *in vivo* using a zebrafish embryo model that showed stronger infection in the eyes and other tissues. Our study provides novel insights into gD receptor independent viral entry pathways and can guide new strategies to enhance the delivery of viral gene therapy vectors or oncolytic viruses.

## Introduction

HSV-1 is prevalent pathogen in various clinical manifestations ranging from common cold sore, gingivostomatitis, herpetic whitlow, corneal herpetic stromal keratitis, genital ulcers, and sometimes more serious complications such as encephalitis and meningitis (Nahmias and Roizman, 1973; Whitley et al., 1998; Whitley and Roizman, 2001). The current model of HSV entry suggests that the virus uses multiple pathways during entry depending on cell types and entry receptors (Spear and Longnecker, 2003; Karasneh and Shukla, 2011; Salameh et al., 2012). HSV-1 entry generally begins with viral attachment to host cells in which HSV glycoproteins B (gB) and C (gC) on the envelope of the virus typically bind to heparan sulfate (HS) on the surface of the host cell (WuDunn and Spear, 1989; Herold et al., 1991; Shieh et al., 1992). This is followed by gD binding to one of its cognate receptors including an intercellular adhesion molecule: nectin −1 or −2 (Geraghty et al., 1998) and a member of the tumor necrosis factor receptor family: herpesvirus entry mediator (HVEM; Montgomery et al., 1996). The virus also uses a modified version of HS known as 3-*O* sulfated heparan sulfate (3-*O*S HS) to bind gD and induce virus cell fusion independent of the known protein receptors (Shukla et al., 1999; Shukla and Spear, 2001). Viral interactions with these receptors initiate fusion between the viral envelope and the membrane of the cell, which also requires participation of two additional HSV glycoproteins gH and gL (Pertel et al., 2001; Spear and Longnecker, 2003). Upon fusion, the nucleocapsid and tegument are released into the cytoplasm where they are transported to the nucleus via microtubules. Our previous studies have shown that HSV entry exploits host cell cytoskeleton via a novel phagocytic uptake in human corneal fibroblasts (CF) cells and that modified 3-*O*S HS plays a significant role in promoting viral entry and spread via F-actin membrane extensions such as filopodia (Clement et al., 2006; Oh et al., 2010; Choudhary et al., 2013).

Because HSV depends on a receptor-mediated entry, CHO cells that naturally lack gD receptors and hence resistant to HSV infections (Montgomery et al., 1996), are a well-known model to study receptor-independent entry of HSV. To make them susceptible, these cells need to be transfected with one of the gD receptors. During one of our transfection experiments, we accidently infected CHO-K1 cells lacking gD receptors with HSV-1 in the presence of cell transfection reagent, Lipofectamine 2000. To our surprise we observed robust viral entry. This was interesting as virus entry was observed in the absence of any gD receptors. Based on this finding, our study investigates a non-receptor mediated HSV entry. It demonstrates a role for HS and the cytoskeleton filaments in gD receptor-independent endocytosis of HSV into naturally resistant CHO-K1 cells. Overall, we provide a new understanding of gD receptor-independent viral entry mechanisms and help generate a new strategy to enhance the delivery of viral gene therapy vectors.

## Materials and methods

Western University of Health Sciences (WUHS) Institutional Animal Care and Use Committee specifically approved this study (IACUC protocol number 026).

### Cell culture and viruses

Wild-type Chinese hamster ovarian-K1 (CHO-K1), reporter CHO-Ig8 cells that expresses β-galactosidase upon viral entry (Montgomery et al., 1996), CHO-745 that lack glycosaminoglycans including HS (WuDunn and Spear, 1989), Vero, HeLa, and primary cultures of human corneal fibroblasts (CF) cells (Clement et al., 2006) were used in this study. The CF was procured from corneal tissue purchased from Illinois eye bank. www.eversightvision.org/illinois/. All CHO cells were grown in Ham's F-12 medium (Gibco/BRL, Carlsbad, CA, USA) supplemented with 10% fetal bovine serum (FBS) and 1% penicillin and streptomycin (P/S, Gibco/BRL). Vero and HeLa cells were grown in Dulbecco's modified Eagle's medium (DMEM) supplemented with 10% FBS and P/S. Primary cultures of human CF were maintained as previously described. The β-galactosidase expressing recombinant HSV-1 (KOS) gL86 and GFP-expressing HSV-1 (K26GFP) were provided by P.G. Spear (Northwestern University, Chicago) and P. Desai (Johns Hopkins University; Desai and Person, 1998). The HSV-1 gL86 is a recombinant virus in which a portion of the “gL” gene was replaced with the *lac Z* gene encoding for the β-galactosidase enzyme (Montgomery et al., 1996). Only upon entry and successful infection, the β-galactosidase enzyme is synthesized and activated. Thus, the activity of this enzyme, using a substrate like ONPG, is measured as an indicator of viral entry. In our experiments the enzymatic activity was measured as optical density (OD) at 410 nm by a spectrophotometer_._

### HSV-1 viral entry assay

CHO-K1, cells were grown in 96 or 6 well-plates to subconfluence and infected with β-galactosidase expressing recombinant gL86 100 pfu/cell using Lipofectamine 2000 (Invitrogen). Uninfected cells in the presence and absence of lipofectamine were used as negative controls. Six hours post-infection (hpi), β-galactosidase assays were performed using *o*-nitrophenyl-D-galactopyranoside (ImmunoPure ONPG; Pierce) or 5-bromo-4-chloro-3-indolyl-_-D-galactopyranoside (X-gal; Sigma). For the soluble substrate, the enzymatic activity was measured at 410 nm using a micro-plate reader (Molecular Devices spectra MAX 190, Sunnyvale, CA). For X-gal assay, the cells were fixed (2% formaldehyde and 0.2% glutaraldehyde) and permeabilized (2 mM MgCl2, 0.01% deoxycholate, and 0.02% nonidet NP-40 Sigma). Finally, 1mL of β-galactosidase reagent (1.0 mg/mL X-gal in ferricyanide buffer) was added to each well and incubated at 37°C for 90 min before the cells were examined using brightfield microscopy under the 20 × objectives (Nikon D-Eclipse-C1). Similar experiments were performed using β-galactosidase expressing CHO-Ig8 cells.

### Immunofluorescence imaging

Cultured monolayers of human corneal fibroblasts (CF) were infected with HSV-1 K26GFP at 50 PFU in serum free media Opti-MEM, and this was followed by fixation of cells at 1 h post-infection using fixative buffer (2% formaldehyde and 0.2% glutaradehyde). The cells were then washed with NaCl/Pi and permeabilized with 2 mM MgCl_2_, 0.01% deoxycholate, and 0.02% Nonidet NP-40 for 20 min. After rinsing with NaCl/Pi, 10 nM rhodamine-conjugated phalloidin (Invitrogen) was added for F-actin staining at room temperature for 45 min. Finally, the cells were washed three times with one NaCl/Pi. Images of immunofluorescent labeled cells were acquired by using a confocal microscope (Nikon D-Eclipse-C1) using the software EZ-C1.

### Scanning electron microscopy (SEM)

Wild-type CHO-K1 and CHO-745 cells were grown in 60 mm plates and infected with HSV-1 (KOS) at 50 pfu/cell for 0, 30, and 60 min at 37°C. Uninfected cells in the presence and absence of lipofectamine were used as negative controls. The cells were then fixed with 2% formaldehyde/ 4% glutaraldehyde in 1 × Dulbecco's phosphate buffer saline (PBS) prior to SEM study. This was followed by fixing cells with 1% osmium tetroxide for 40 min. Dehydration was done in an increasing order (25–100%) of ethanol treatment at 5 min each, respectively. Hundred percentage ethanol was repeated to ensure dehydration. Cover slips were removed from dishes and mounted on aluminum studs previously cleaned with 100% ethanol. Cover slip edges were painted with colloidal silver for conduction and dried in a Critical Point Dryer (Samdri-780A). Samples were then coated with gold using a Sputter Coater (Hummer VI-A) for 2 min. Samples were viewed using a Hitachi S-2700 Scanning Electron Microscope (SEM). Images were captured at 1000–5000x using 4Pi Revolution image capture system.

### Western blots

Heparan sulfate (HS) positive CHO-K1 and HS negative CHO-745 cells were infected for 3 h at 37°C with HSV-1 gL86 virus at 10 PFU/cell in presence and absence of lipofectamine (8 μg/mL). Cells were lysed in radioimmunoprecipitation buffer (RIPA, Sigma) with Proteinase and Phosphatase (Halt, Pierce Biotechnology) and electrophoresed on a 4–12% Bis-Tris Gel (NuPage). A PVDF membrane (Novex) was used for transfer. After initial non-specific blocking in 5% non-fat milk, the membrane was then incubated with primary (VP16 mouse monoclonal, Santa Cruz; GAPDH rabbit polyclonal, Santa Cruz) and secondary HRP-conjugated antibodies (Jackson). The membrane was then developed (ECL, Pierce) and visualized (ImageQuant LAS 4000, GE Healthcare).

### Zebrafish experiments

Usage of Zebrafish (ZF) embryo experiment was conducted under approved protocol (IACUC/026) by WUHS, Pomona, California. WIK strain of 1 day old Zebrafish embryos were obtained from ZFIN. On day 3 the zebrafish embryos were infected with a mixture containing 10^8^ HSV in 10 μg/mL lipofectamine (10 μg/mL) in 96 well-dishes. In parallel experiments, zebrafish embryos were pre-treated with heparinase I and II (1U/ml for 5 h) or mock treated before HSV-1 infection. Addition of the soluble substrate [o-nitrophenyl-β-D-galactopyranoside (ONPG, ImmunoPure, Pierce; 3 mg/ml)] in the culture medium followed by fluorescence measurement led to the generation of dose–response curves for HSV-1 infected zebrafish embryos. The enzymatic activity was measured at 410 nm using a micro-plate reader. For X-gal assay, after 24 h post-infection, the embryos were fixed (2% formaldehyde and 0.2% glutaraldehyde) overnight at 4°C, permeabilized (2 mM MgCl2, 0.01% deoxycholate, and 0.02% nonidet NP-40 (Sigma) for 8 h at 4°C and then 1 mg/mL X-gal in ferricyanide buffer was added to the embryos and left overnight at 37°C. The embryos were then imaged the next day.

### Statistics

The data presented in each experiment are the means of triplicate measures and are representative of three independent experiments. Significant differences were calculated using *t*-test (Analysis of Variance). *P* < 0.05 was considered statistically significant.

## Results

### HSV-1 entry is enhanced by lipofectamine in the absence of glycoprotein D (gD) receptors and is viral strain independent

**Figure 1:**
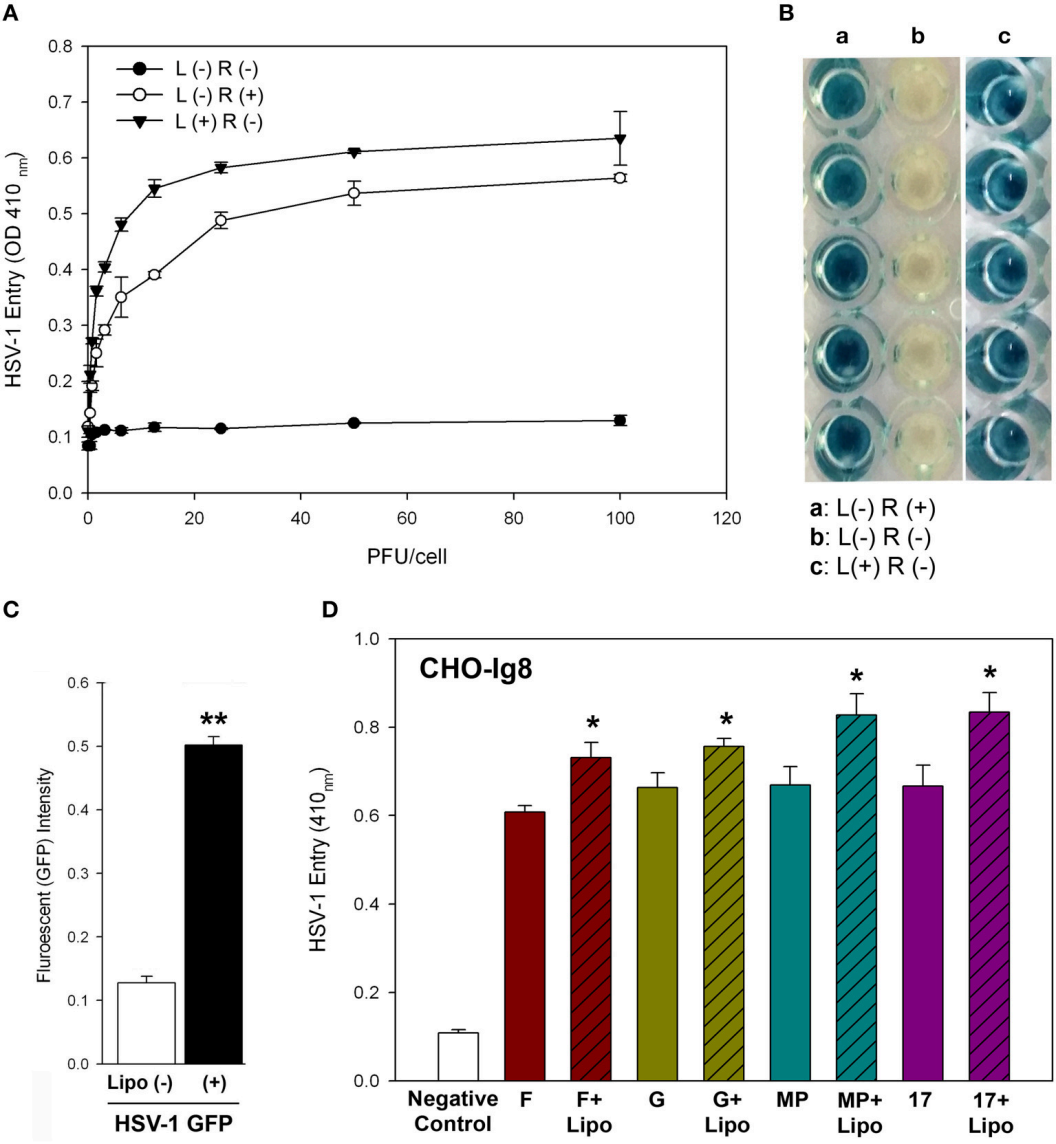
**Lipofectamine-HSV-1 entry into receptor negative CHO-K1 cells. (A)** ONPG virus entry in CHO-K1 cells infected with gL86 in the presence (+) and absence (–) of lipofectamine (4 μg/mL) quantified at 6hpi. **(B)** Stained images of X-gal in CHO-K1 cells infected with gL86 in the presence (+, panel c) and absence (–, panel b) of lipofectamine (4 μg/mL). **(C)** Wild type CHO-K1 cells were infected with K26-GFP with and without lipofectamine (4 μg/mL) and fluorescence intensity was quantified 3hpi. Asterisks indicate significant difference from the uninfected control (*P* < 0.05, *t*-test); error bars represent *SD* (*n* = 4). **(D)** 3-*O*ST-3 isoform expressing CHO Ig8 cells were infected with the indicated strains in the presence (bars with cross line) and absence (plain bars) of lipofectamine and virus entry was measured by ONPG. The data shown are the means of triplicate measures and are representative of three independent experiments. Asterisks indicate significant difference from the uninfected control (*P* < 0.05, *t*-test); error bars represent *SD* (*n* = 3). L, Lipofectamine; R, Receptor.

In order to confirm that lipofectamine helps facilitate HSV-1 infection, an entry assay was performed using the resistant CHO-K1 cell line (Montgomery et al., 1996). We used commercially available cationic liposome (Lipofectamine 2000) in our experiments. HSV-1 entry into the cells was determined by using β-galactosidase expressing HSV-1 reporter virus (gL86). Expression levels of β-galactosidase are induced by HSV infection and therefore it can be used as a measureable indicator of viral entry (Montgomery et al., 1996). Quantification and visualization of β-galactosidase levels by using two different substrates: ONPG and X-gal, show that HSV-1 gL86 pre-incubation with lipofectamine (L) resulted in higher levels of HSV-1 entry in receptor (R) negative CHO-K1 cells (Figures 1A,B). Similarly, enhanced viral entry was also noted in wild-type CHO-K1 cells when Green fluorescent protein (GFP)-tagged HSV-1 virions (K26GFP; Desai and Person, 1998) was in a mixture with lipofectamine (Figure 1C). To check if non-receptor mediated viral entry is HSV strain dependent, we tested the ability of lipofectamine to enhance viral entry of different strains of HSV-1/-2 (F, G, MP, and 17 strains). Here we used CHO Ig8 cells that express β-galactosidase upon viral entry (Montgomery et al., 1996). The HSV strains were pre-incubated with lipofectamine and subsequently infection was performed. The results from this experiment showed that lipofectamine enhanced entry of different HSV strains in a dosage dependent manner as evident by ONPG assay (Figure 1D). It has been previously shown that liposome encapsulation of retrovirus and hepatitis D virus allows efficient infection in resistant cell lines (Innes et al., 1990; Bichko et al., 1994). A similar effect is likely seen with lipofectamine/HSV mixture.

### Presence of lipofectamine-HSV-1 mixture further enhances entry in natural target cells

**Figure 2:**
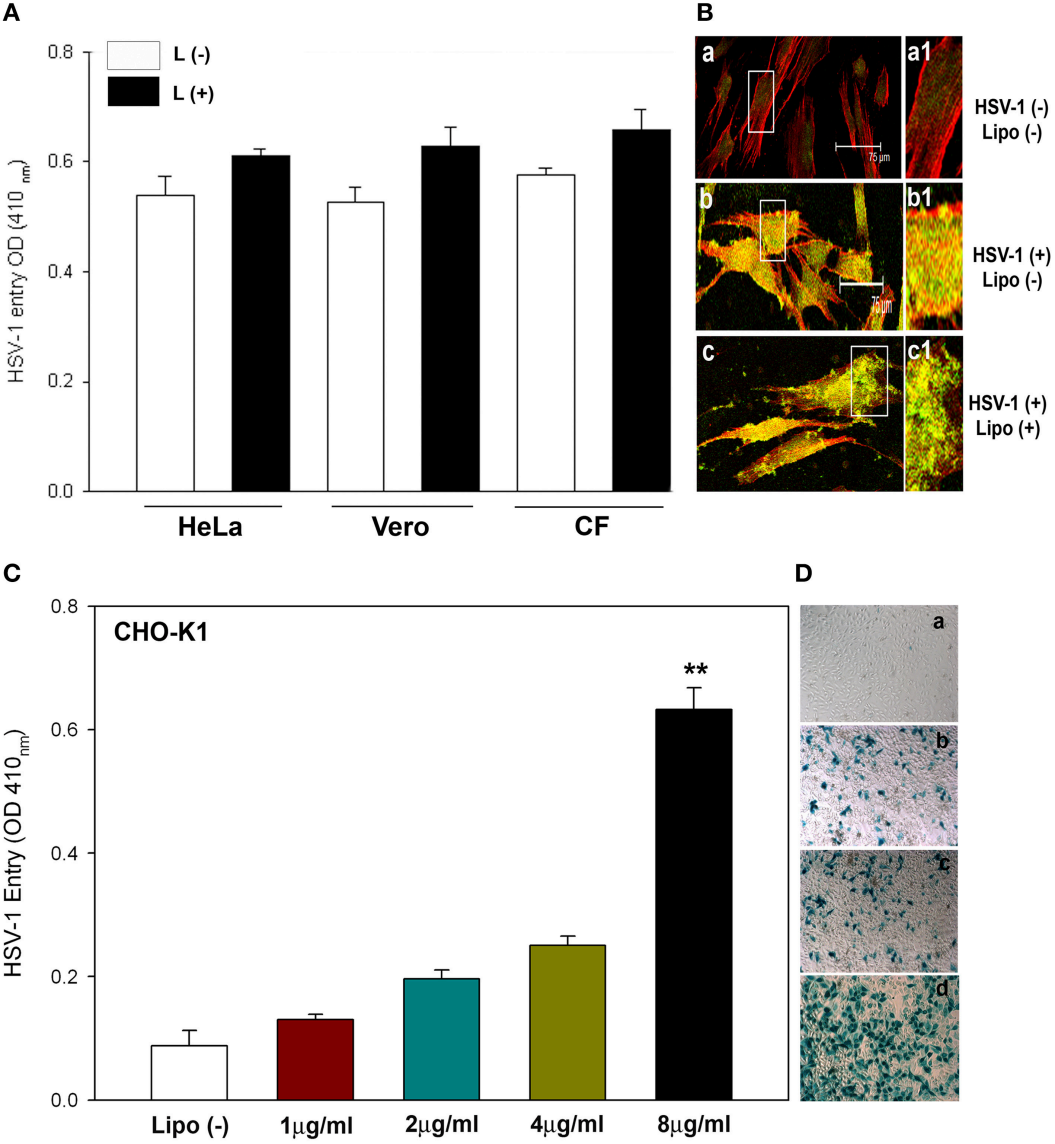
**Enhanced effect on HSV-1 entry in naturally susceptible cells in the presence of lipofectamine. (A)** The indicated cells were plated in 96 well-plates and infected with gL86 virus in the presence (black bars) and absence of (white bars) lipofectamine. The viral entry was measured by ONPG assay 6hpi. L, Lipofectamine **(B)** Human CF, stained with actin (shown as red), was infected with K26-GFP in the presence (panel c) and absence (panel b) of lipofectamine. Uninfected and lipofectamine untreated CF served as negative control (panel a). The boxed regions in panel a–c are highlighted as a1–c1. The data shown are the means of triplicate measures and are representative of three independent experiments. **(C)** Lipofectamine concentration dictates HSV-1 entry. CHO-K1 cells in 96 well-plates were infected with gL86 along with the indicated concentrations of lipofectamine. Entry was quantified 6hpi by ONPG assay. Asterisks indicate significant difference from the uninfected control (*P* < 0.05, *t*-test); error bars represent SD (*n* = 3). **(D)** Virus entry by X-gal assay in CHO-K1 cells infected with gL86 with 2 μg/mL (panel b), 4 μg/mL (panel c), and 8 μg/mL (panel d) of lipofectamine and without lipofectamine (panel a). The data shown are the means of triplicate measures and are representative of three independent experiments.

We next evaluated the ability of lipofectamine to enhance HSV-1 entry into naturally permissive or target cell lines. Confluent monolayers of HeLa, Vero, and primary cultures of human CF were plated and infected with HSV-1(gL86) in the presence and absence of lipofectamine and the entry of HSV-1 were measured using the assay mentioned above. β-galactosidase expression levels were higher in the cells that were treated with lipofectamine (black bar) than the parallel untreated control (white bar). The entry was not significantly higher as HeLa, Vero, and CF are known to express gD receptors and are already highly susceptible to HSV-1 infection (Tiwari et al., 2006; Figure 2A). In order to obtain more direct and visual evidence of lipofectamine-mediated HSV-1 entry in CF cells, K26GFP was pre-incubated with lipofectamine to infect cultured CF, and fluorescence microscopy was used to visualize the virions (Figure 2B). The GFP-punctate dots with high intensity were noticed when HSV-1 was allowed to infect in the presence of lipofectamine (Figure 2Bc), compared to GFP-HSV-1 in the absence of lipofectamine (Figure 2Bb). This suggests that lipofectamine-virus mixture were equally effective in delivering HSV-1 into susceptible cells expressing gD receptors and the system of introducing lipofectamine-virus mixture into susceptible cells were not interfered by the presence of gD receptors.

### Lipofectamine-HSV-1 mixture mediated entry is concentration dependent

To quantitatively demonstrate the effectiveness of lipofectamine in allowing HSV-1 entry into resistant CHO-K1 cells, we incubated different concentrations (from 1.0–8.0 μg/mL) of lipofectamine with gL86 for 15 min at room temperature. The PBS treated gL86 was considered as a control for comparison. Maximum viral entry, via ONPG and X-gal assays, was detected at a higher concentration (8.0 μg/mL) of lipofectamine (Figures 2C,Dd). Again, HSV-1 without lipofectamine failed to infect CHO-K1 cells (Figure 2Da). Similar concentration (8.0–10 μg/mL) of lipofectamine has been reported to mediate ecotropic murine leukemia virus and hepatitis D virus infections (Innes et al., 1990; Bichko et al., 1994).

### Cell surface heparan sulfate plays a critical role during lipofectamine-mediated gD-receptor-independent HSV-1 entry

**Figure 3:**
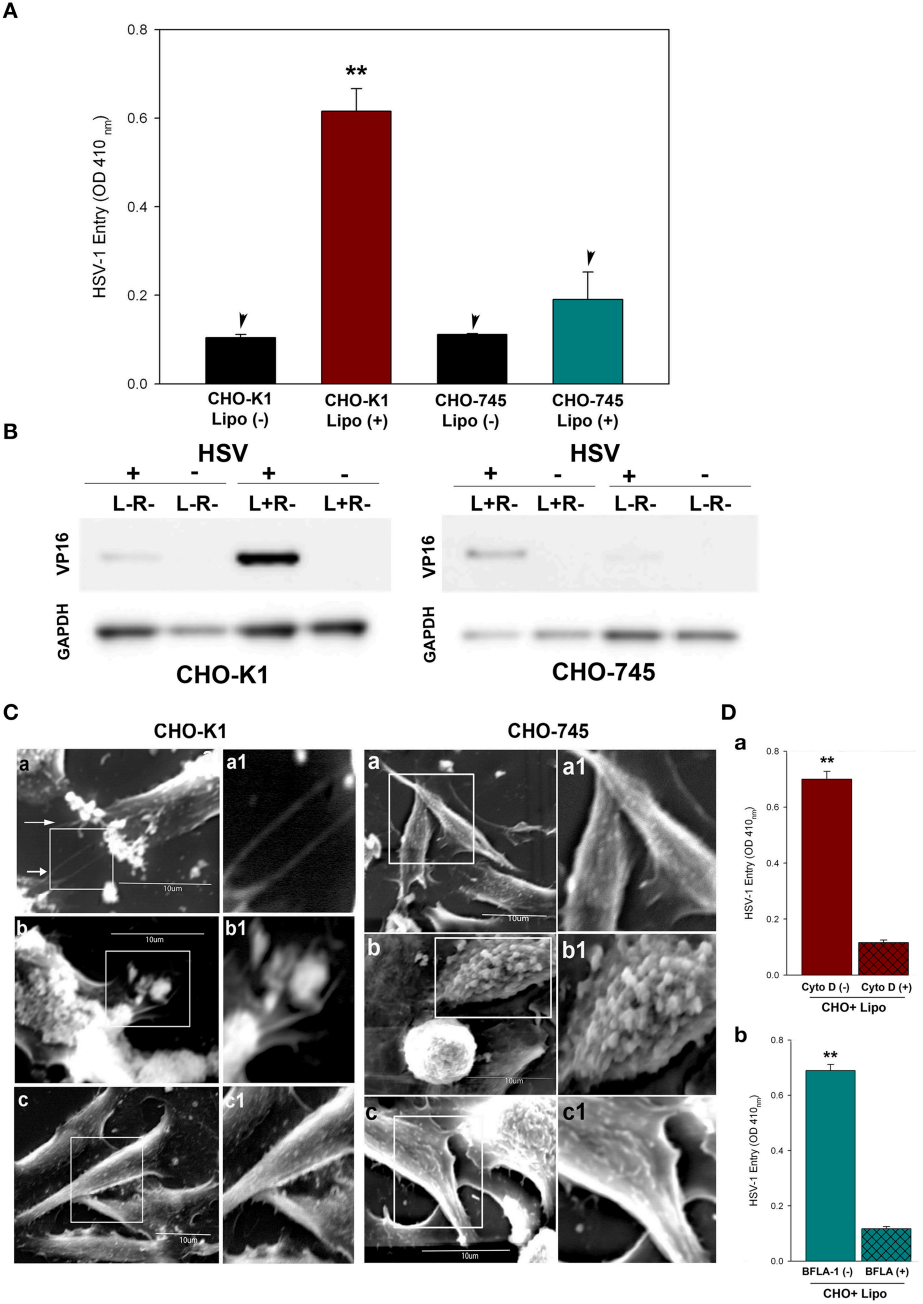
**Significance of heparan sulfate (HS) in lipofectamine mediated HSV-1 uptake. (A)** Virus entry in CHO-K1 and HS deficient CHO-745 cells infected with gL86 in the presence and absence of lipofectamine (4 μg/mL) was determined by ONPG. Asterisks indicate significant difference from the CHO-K1 cells infected in absence of lipofectamine (*P* < 0.05, *t*-test); error bars represent *SD* (*n* = 3). While CHO-745 cells infected with HSV-1 in presence and absence of lipofecatine had no significant differences. **(B)** VP-16 levels compared between CHO-K1 and HS negative (CHO-745) cells via western blot (L, Lipofectamine; R, Receptor). **(C)**. Scanning electron microscopy (SEM) performed on CHO-K1 cells infected with HSV-1 along with lipofectamine shows filiopodia (panel a) and HSV-1 uptake (panel b), while HSV-1 without lipofectamine infected cells show smooth surface (panel c). The boxed regions in panel a–c are highlighted as a1–c1. In parallel lipofectamine mixture with HSV-1 had little or no actin cytoskeleton activity in CHO-745 cells (panel a,b). Similarly no filopodia were noticed in HSV-1 infected CHO-745 cells in absence of lipofectamine (panel c). The boxed regions in panel a–c are highlighted as a1–c1. The data shown are the means of triplicate measures and are representative of three independent experiments. **(D)** Host cell cytoskeleton is critical during uptake of lipofectamine-HSV-1 mixture in absence of gD receptors. **(D)** Panel a: Cultured monolayers of CHO-K1 cells were pre-treated with the 0.5 μg/mL actin depolymerizing agent Cytochalisin D (Cyto D; panel a) before infecting with gL86 along with lipofectamine. Cells treated with 1 × PBS treated cells were used as a control. Viral entry was measure by ONPG assay. Panel b: Cells were pre-treated with 0.02 μM Bafilomycin A1 (BFLA-1) and then infected with gL86. Viral entry was measured using ONPG assay. The data shown are the means of triplicate measures and are representative of three independent experiments. Asterisks indicate significant difference from the Cyto-D/BFLA-1 treated control (*P* < 0.05, *t*-test); error bars represent SD (*n* = 3).

We further probed the mechanism non-receptor mediated entry by investigating the role of cell surface HS which are highly expressed on CHO-K1 cells. It is well-documented that HS is a negatively charged cell surface attachment receptor that virtually all human herpesviruses recognize during initial attachment to cells (Shukla and Spear, 2001) and has a role in disease manifestations (Ferro, 2013). The significance of HS in absence of gD receptor is well-recognized as CHO-K1 cells allow sufficient viral binding/attachment but not entry because of lack of gD-receptor, while CHO mutant cells defective in HS biosynthesis (CHO-745) do not allow viral binding or attachment (Shukla et al., 1999). To determine whether presence of anionic HS assists the delivery of cationic lipofectamine-virus mixture into cells, we infected both CHO-K1 and CHO-745 cells with HSV-1 in the presence and absence of lipofectamine. The untreated HSV-1 was kept as negative control for both types of cells. Entry was quantified by ONPG assay and the levels of viral tegument protein: VP16 (Heine et al., 1974; Weinheimer et al., 1992) was assessed by western blot. Lipofectamine mediated HSV entry was prominent in wild-type CHO-K1 cell but not in CHO-745 cells (Figures 3A,B) suggesting the role of HS involved in viral entry mediated by lipofectamine.

To further verify whether lipofectamine mediated entry was HS-dependent, high resolution scanning electron microscopy (SEM) was performed. HS expressing wild-type CHO-K1 cells and HS deficient CHO-745 cells were infected with HSV-1 in the presence of lipofectamine and processed for SEM. The SEM images demonstrated the presence of large numbers of virus particles attached to F-actin membrane structures including filopodia, which are induced upon infection and express higher amounts of HS (Oh et al., 2010; Figures 3Ca,Cb), while uninfected CHO-K1 cells had a relatively smooth surface (Figure 3Cc). In contrast, virus-lipofectamine mixture were adhered and clumped on the cell membrane of CHO-745 cells (Figures 3Cb,Cc) as no or very little outgrowth of filopodia was noticed in both infected and uninfected CHO-745 cells (Figure 3Ca). These results show that HS may play a significant role in HSV-1 entry in the absence of gD-receptor.

### Endocytosis of lipofectamine-virus mixture during entry exploits actin-cytoskeleton including filopodia

As lipofectamine and HSV mainly enter cells by endocytosis (Nicola et al., 2003; Cui et al., 2012), we speculated that the lipofectamine-HSV mixture is also being endocytosed. It has been reported that HSV entry through endocytosis leads to a change in arrangement of cytoskeleton elements (Lyman and Enquist, 2009). As discussed above, we found enhancement of filopodia formation and the presence of virions on the filopodia from SEM imaging in CHO-K1 cells. Based on this observation, we predicted that actin filaments can be beneficial to non-receptor mediated entry. To demonstrate this, viral entry assay was performed in the presence of an F-actin depolymerizer. CHO-K1 cells were pre-treated with Cytochalasin D (Cyto D) prior to infection. It was postulated that pre-treatment of Cyto-D would have more negative effect provided the actin-based membrane protrusions (such as filopodia) played a role in the attachment and entry of lipofectamine-virus mixture. As predicted, actin depolymerizing agent Cyto-D negatively affected virus entry (Figure 3D, Top panel). The expected loss of filopodia due to Cyto-D treatment in these experiments correlates well to our prior knowledge that filopodia plays a significant role during HSV-1 entry (Oh et al., 2010). Next, we examined the role of pH dependence in non-receptor mediated entry. The rationale was based on the fact that lipofectamine-virus mixture might be taken in an endosomal vesicle which has an acidic pH (Geisow and Evans, 1984). Thus, effects of lysosomotropic agent (bafilomycin A1; BFLA-1) that are capable of interfering with vesicular acidification (Lukacs et al., 1990) were tested for their effects on virus entry. Lipofectamine-virus mixture showed significantly low viral entry in the presence of BFLA-1 reinforcing the point that pH plays an important role in non-receptor mediated virus entry (Figure 3Db). Taken together, it can be implied that lipofectamine-virus mixture enters through endocytosis and depends on actin filaments for the uptake and low pH for a successful entry.

### Lipofectamine-virus mixture promotes HSV infection in zebrafish embryo model

**Figure 4:**
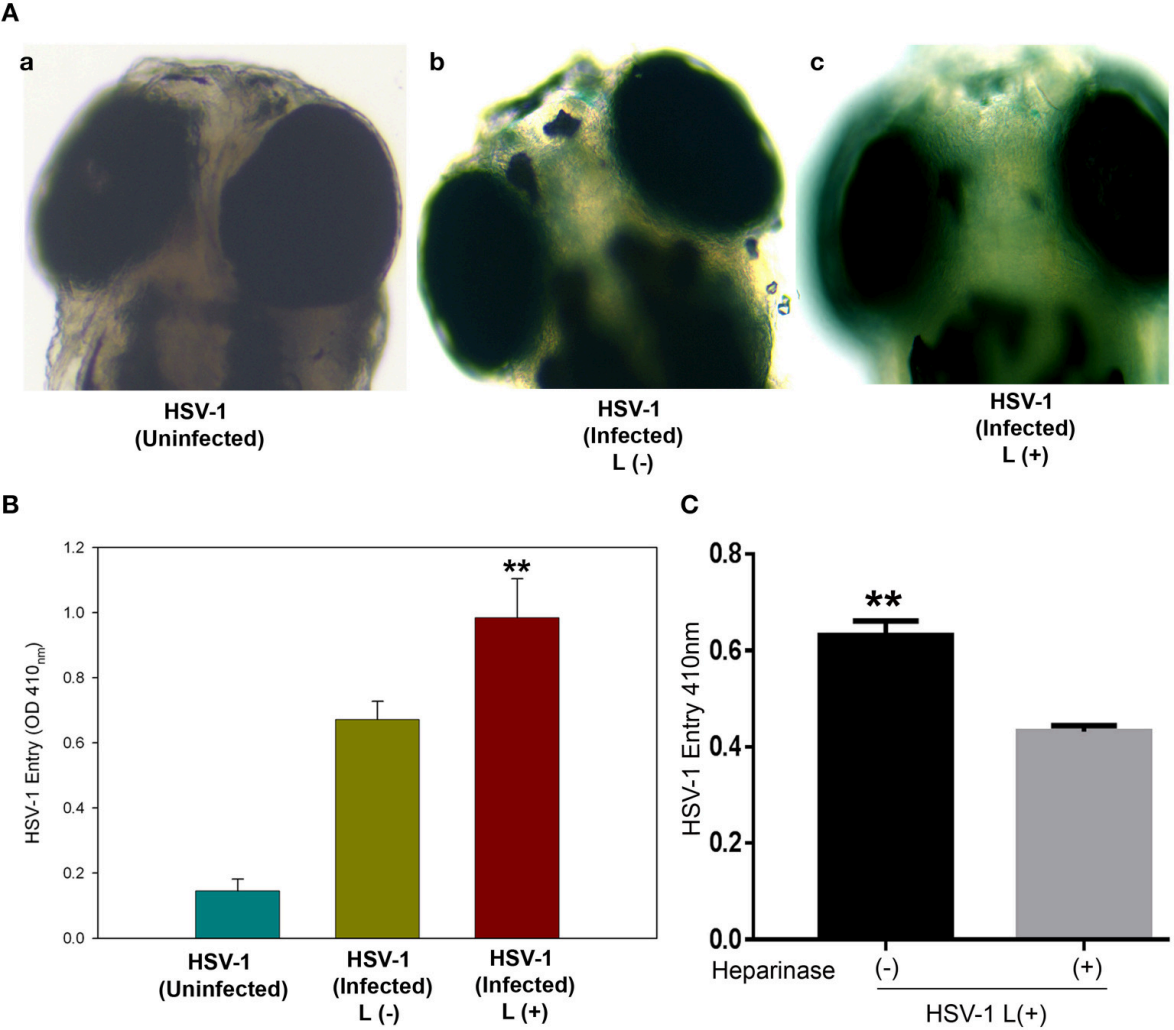
**Significance of lipofectamine HSV-1 mixture uptake in Zebrafish embryo model**. WIK strain of 3 day old Zebrafish embryo was infected with 10^8^ HSV in presence (panel c) and absence (panel b) of lipofectamine (10 μg/mL). **(A)** shows 24hpi X gal staining in Zebrafish embryos, while **(B)** indicates ONPG assay conducted with Zebrafish embryos in the presence and absence of lipofectamine. Asterisks indicate significant difference from the uninfected control (*P* < 0.05, *t*-test); error bars represent *SD* (*n* = 3). L: Lipofectamine. **(C)**: pre-treatment of Zebrafish embryos with heparinase I and II (1U/ml) reduce lipofectamine (L) mediated HSV-1 entry compare to heparinase untreated embryos. Reporter virus based HSV-1 entry assay was conducted by using ONPG assay. The data shown are the means of triplicate measures and are representative of three independent experiments. Asterisks indicate significant difference from the heparinase untreated control (*P* < 0.05, *t*-test); error bars represent *SD* (*n* = 3).

Finally, we tested the *in vivo* significance of lipofectamine-virus mixture in a Zebrafish (ZF) infection model (Burgos et al., 2008). Using 3 day old ZF embryos, HSV-1 infection with and without lipofectamine was performed. HSV entry in ZF embryos were assessed by ONPG and X-gal assays after 18 h post-infection. A high intensity blue staining of ZF embryos were observed in virus-lipofectamine treated group compared to virus alone infected embryos (Figures 4Ab,Ac) indicating that more virus has entered. The mock infected ZF embryo remained colorless following X gal staining (Figure 4Aa). A similar pattern of HSV entry was observed and quantified in zebrafish embryos by ONPG assay (Figure 4B). Interestingly, pre-treatment of ZF-embryos with heparinase I and II (1 U/mL) reduces lipofectamine mediated virus HSV-1 entry (Figure 4C). Thus, it was clear that lipofectamine-mediated virus infection can be achieved in live animal models.

## Discussion

To summarize, our study, for the first time, describes how HSV-1 gets into cells by a non-receptor mediated endocytosis, meaning that virus entry and infection can occur without the presence of gD receptors. Receptor mediated endocytosis requires the presence of at least one of the gD receptors (Shukla and Spear, 2001) for viral entry and infection to occur. However, using a lipofectamine-virus mixture, we show that in gD receptor lacking CHO-K1 cells, the virus can successfully enter and infect without the need of gD receptors (Figure 1). We compared this mode of entry in natural target cells such as HeLa and primary corneal fibroblasts. Though entry levels were not significant (as these cells naturally express gD receptors), higher entry levels in the presence of lipofectamine were observed consistently in all natural target cells (Figure 2). Also, the presence of natural gD receptors in these cells did not interfere with the entry of lipofectamine-virus mixture suggesting that HSV-1 entry in the presence of lipofectamine can still use gD receptors. We also found that lipofectamine-virus mixture is not virus strain dependent (Figure 1D), and can be used to enhance entry in live animal models, suggesting the universal nature of lipofectamine-mediated entry.

Previous studies show the importance of the rearrangement of cytoskeleton elements and pH dependence for endocytosis (Nicola et al., 2003; Lyman and Enquist, 2009; Cui et al., 2012). Keeping this into consideration, we show that non-receptor mediated entry requires actin filaments and low pH for a successful HSV-1 entry and blocking either one of these factors results in loss of viral entry indicating that lipofectamine-HSV mixture uses endocytosis as a mode of entry. However, an intriguing question arises at this point: if the virus does not use gD receptor, how does it lose its coat proteins, get endocytosed and become infectious? One of the plausible assumptions could be that low pH of endosomes may play a role in the virus becoming infectious. Low pH is known to bring about conformational changes in glycoprotein B (gB), which are much favorable for fusion with a vesicular membrane and may not require a gD receptor (Dollery et al., 2010). More specifically, some gB receptors such as non-muscle myosin IIA could facilitate the process (Arii et al., 2010). It is also possible that lipofectamine by its membrane permeability property may itself expose the fusogenic domain of gB without requiring the gD/receptor mixture to trigger the process. This mixture could thus be potentially used for delivering a gD null virus for vaccine development and functional studies. Future studies will determine the exact mechanism by which the capsid is released into the cytosol.

There is enough evidence to suggest that HS plays an important role in HSV pathogenesis (Shukla and Spear, 2001). In this study we demonstrated a novel role that HS plays in non-receptor mediated entry. The presence of HS on cell surface clearly enhances entry (Figure 3). The need for HS could be crucial for Lipofectamine-virus delivery due to fact that HS is negatively charged and may directly interact with cationic liposomes. Entry into animal models such as ZF also requires HS (Antoine et al., 2014) and therefore, the fact that Lipofectamine enhances entry *in vivo* could be used to identify the tissues which may be more susceptible due to the presence of HS. While zebrafish has been shown to support entry of HSV and other viruses, more tedious ways such as microinjection is normally required to initiate viral entry in this model (Burgos et al., 2008; Antoine et al., 2014). Our study also demonstrates a much easier way to facilitate HSV-1 entry in zebrafish (Figure 4) and does not require sophisticated microinjection equipment. Interestingly, we observed a higher X-gal staining in the eye and other tissues in the virus-lipofectamine treated group compared to virus alone infected embryos (data not shown). Although more studies are needed to outline the actual mechanism by which lipofectamine-virus mixture is entering and infecting cells and how the diversity of HS proteoglycans can affect this, our demonstration of the HSV uptake in the absence of gD receptors has given a platform to look beyond the role of gD receptors and discover new receptors or components and learn about their role in viral entry and transport. In addition, because the roles of gD in nuclear viral egress (Johnson and Baines, 2011; Johnson et al., 2011) and cell-to-cell spread (Pertel et al., 2001; Akhtar and Shukla, 2009; Karasneh and Shukla, 2011) are well-established, the lipofectamine-virus mixture may be utilized to shed light on the possible roles of gD receptors in the above mentioned events. Further, role of other HSV-1 glycoproteins such as gK which is known to be involved in virus spread and alterations in viral receptor expression in the eye (Allen et al., 2014), needs to be investigated during HSV-1-liposome mixture mediated infection in zebrafish ocular model.

## Author contributions

LB and DJ equally contributed to the work. KJ and VT did the zebrafish experiments. LB and JT did the Electron Microscopy studies. VT designed the study and analyzed the data. DJ, DS, and VT wrote the manuscript. LB, JT, DJ, DS, KJ, and VT read, corrected and approved the final manuscript.

## Funding

This investigation was supported by internal Instructional grant support (grant number N12587) to VT from WUHS, Pomona, CA, and grants from National Institutes of Health 1R15 AI088429-01A1 and R21 AI105573.

### Conflict of interest statement

The authors declare that the research was conducted in the absence of any commercial or financial relationships that could be construed as a potential conflict of interest.
